# RIPK3 Is Largely Dispensable for RIG-I-Like Receptor- and Type I Interferon-Driven Transcriptional Responses to Influenza A Virus in Murine Fibroblasts

**DOI:** 10.1371/journal.pone.0158774

**Published:** 2016-07-08

**Authors:** Shoko Nogusa, Michael J. Slifker, Justin P. Ingram, Roshan J. Thapa, Siddharth Balachandran

**Affiliations:** 1 Blood Cell Development and Function Program, Fox Chase Cancer Center, Philadelphia, Pennsylvania, United States of America; 2 Department of Bioinformatics and Biostatistics, Fox Chase Cancer Center, Philadelphia, Pennsylvania, United States of America; Johns Hopkins School of Medicine, UNITED STATES

## Abstract

The kinase RIPK3 is a key regulator of cell death responses to a growing number of viral and microbial agents. We have found that influenza A virus (IAV)-mediated cell death is largely reliant on RIPK3 and that RIPK3-deficient mice are notably more susceptible to lethal infection by IAV than their wild-type counterparts. Recent studies demonstrate that RIPK3 also participates in regulating gene transcription programs during host pro-inflammatory and innate-immune responses, indicating that this kinase is not solely an inducer of cell death and that RIPK3-driven transcriptional responses may collaborate with cell death in promoting clearance of IAV. Here, we carried out DNA microarray analyses to determine the contribution of RIPK3 to the IAV-elicited host transcriptional response. We report that RIPK3 does not contribute significantly to the RLR-activated transcriptome or to the induction of type I IFN genes, although, interestingly, IFN-β production at a post-transcriptional step was modestly attenuated in IAV-infected *ripk3*^*-/-*^ fibroblasts. Overall, RIPK3 regulated the expression of <5% of the IAV-induced transcriptome, and no genes were found to be obligate RIPK3 targets. IFN-β signaling was also found to be largely normal in the absence of RIPK3. Together, these results indicate that RIPK3 is not essential for the host antiviral transcriptional response to IAV in murine fibroblasts.

## Introduction

The kinase RIPK3 is essential for a form of programmed necrosis termed necroptosis. A whole-genome screen for mediators of RIPK3-mediated necroptosis identified several regulators of antiviral and antimicrobial innate immunity [1], and numerous studies have since shown that RIPK3 and the related kinase RIPK1 control necrotic and inflammatory outcome after infection by certain bacteria and viruses, as well as after exposure of cells to select pathogen-associated molecular patterns (PAMPs) and cytokines [2–4]. Under certain conditions, such as when caspases are inhibited or the adaptor protein FADD is disabled, stimulation of cells with these agonists induces phosphorylation-driven assembly of RIPK1 and RIPK3 into a higher-order molecular complex called the necrosome [5]. Necrosome assembly promotes RIPK3-driven phosphorylation of the pseudokinase MLKL, which then inserts into lipid membranes to disrupt cellular integrity and trigger necrotic death [2]. Complicating this simplistic view of RIPK1/3-mediated MLKL-dependent necrotic cell death, recent studies have revealed that both RIPK1 and RIPK3 participate in activation of inflammasomes and induction of pro-inflammatory gene transcription pathways independently of their cell death activities and often without requirement for their enzymatic activity [3,6–8]. Whether RIPK3 contributes to antiviral gene transcription programs remains unknown.

The primary antiviral gene transcriptional response to an IAV infection is activated by the RIG-I-like Receptor (RLR) class of cytoplasmic RNA helicases [9,10]. RLRs sense IAV RNA and engage the adaptor protein MAVS (IPS-1/VISA/Cardif) to induce a powerful antiviral and pro-inflammatory gene expression program orchestrated primarily by the IRF and NF-κB classes of transcription factor [9,10]. Among the genes directly activated by RLRs are members of the type I interferon (IFN, predominantly α/β) family. In a long-standing model, the early-phase type I IFNs IFN-β and IFN-α4 are rapidly produced by the infected cell and act on the producing cell itself, as well as on surrounding uninfected cells, to stimulate the expression of a further set of several hundred genes, including the gene encoding the transcription factor IRF-7 [11]. IRF-7 is a short-lived protein whose *de novo* synthesis is necessary for the expression of ‘late-phase’ IFN-α sub-types (i.e. non-α_4_s) in infected cells [12,13]. Thus, the RLR transcriptional response is thought to play out in two parts: a primary wave of transcription that is directly triggered by RLR activation, and a second phase that is regulated by type I IFNs and IFN-induced IRF-7 [11–13]. Together, the biphasic RLR response is critical for protection against most classes of RNA virus, including IAV.

We have found that RIPK3 is an essential orchestrator of the cell death response to IAV in fibroblasts and airway epithelial cells [14]. Mice lacking RIPK3 are significantly more susceptible to IAV replication and spread following pulmonary infection, and consequently succumb to this virus more readily than do their wild-type counterparts [14]. As RLR signaling and type I IFNs are also essential to limiting IAV spread and lethality, and as RIPK3 was previously reported to participate in pro- inflammatory gene transcription programs [3,6–8], we sought to identify the contribution of RIPK3 to the RLR transcriptome following infection with influenza A virus (IAV). We report here that RIPK3 is dispensable for RLR-driven transcriptional activation of type I IFNs and other antiviral genes in murine fibroblasts, but plays a modest and selective role in post-translational production of IFN-β upon infection by IAV.

## Materials and Methods

### Cells, viruses and reagents

*Ripk3*^*-/-*^ [15], *mavs*^*-/-*^ [16] and *ifnar1*^*-/-*^ [17] MEFs were generated from E14.5 embryos under a protocol (#11–17) approved by the Fox Chase Cancer Center Animal Care and Use Committee. Pregnant mice from timed crosses or each genotype were euthanized by CO_2_ inhalation prior to isolation of embryos for generation of MEFs. MEFs were cultured in high-glucose Dulbecco’s Modified Eagle Medium (DMEM) supplemented with 10–15% heat-inactivated fetal bovine serum (Hyclone) and antibiotics, and used within five passages in all experiments. PR8-ΔNS1 was grown in Vero cells as previously described [18] and provided by Paul Thomas (St. Jude children’s Hospital, TN). RIPK3 inhibitor (GSK’872) from GlaxoSmithKline has been described before [19]. Poly(I:C) (Invivogen) and murine interferon-β (PBL interferon source) were purchased from the indicated commercial sources. Antibodies for immunoblot analysis of β-actin (Sigma), ISG15 (Cell Signaling), PKR (Santa Cruz), RIG-I (Enzo), RIPK3 (ProSci), and STAT1 (BD Transduction labs) were obtained from the indicated commercial sources and used in immunoblots as described previously [20]. ELISA kits for the detection of IFN-β and IFN-α were purchased from PBL and used as specified by the manufacturer. For qRT-PCR, mRNA was isolated from poly(I:C) transfected cells by phenol-chloroform extraction, and a one-step reaction was performed using ABI PRISM 7000 Sequence Detection System with their validated primer-probe mix and PCR master mix (Applied Biosystems).

### Microarray analyses

MEFs (2x10^6^ cells/condition in duplicate) were infected with PR8-ΔNS1 (m.o.i. = 1). Total RNA was isolated in TRIzol reagent (Invitrogen) and purified using the RNeasy kit (Qiagen). RNA purity and integrity were evaluated by 2100 Bioanalyzer (Agilent) and Nanodrop 1000 (Nanodrop Technologies). 200ng total RNA was amplified and labeled using Agilent Low Input QuickAmp labeling kit following the manufacture’s protocol. 1.65μg of Cy-3 labeled cRNA target was hybridized onto Agilent 4x44k whole genome arrays for 17 h at 65°C and washed according to the manufacturer’s instructions (Agilent). Hybridized slides were scanned at 5μm resolution on Agilent scanner 2505B and fluorescent intensities of hybridization signals were extracted using Agilent Feature Extraction software (v9.5.3). Identification of differentially expressed genes was performed with the *limma* package [21] implemented in the R/Bioconductor platform [22]. Heatmaps were generated using in-house code written in R.

### Statistics

Graphs were generated using GraphPad Prism 4 software. Error bars represent +/- standard deviation of three replicates of a representative experiment. Each experiment was repeated at least two times, with similar results. Student’s t-test and two-way ANOVA were employed to analyze the differences between sets of data. *p* <0.05 was considered statistically significant.

## Results

### Role of RIPK3 in RLR-induced transcriptional responses to IAV in MEFs

**Fig 1:**
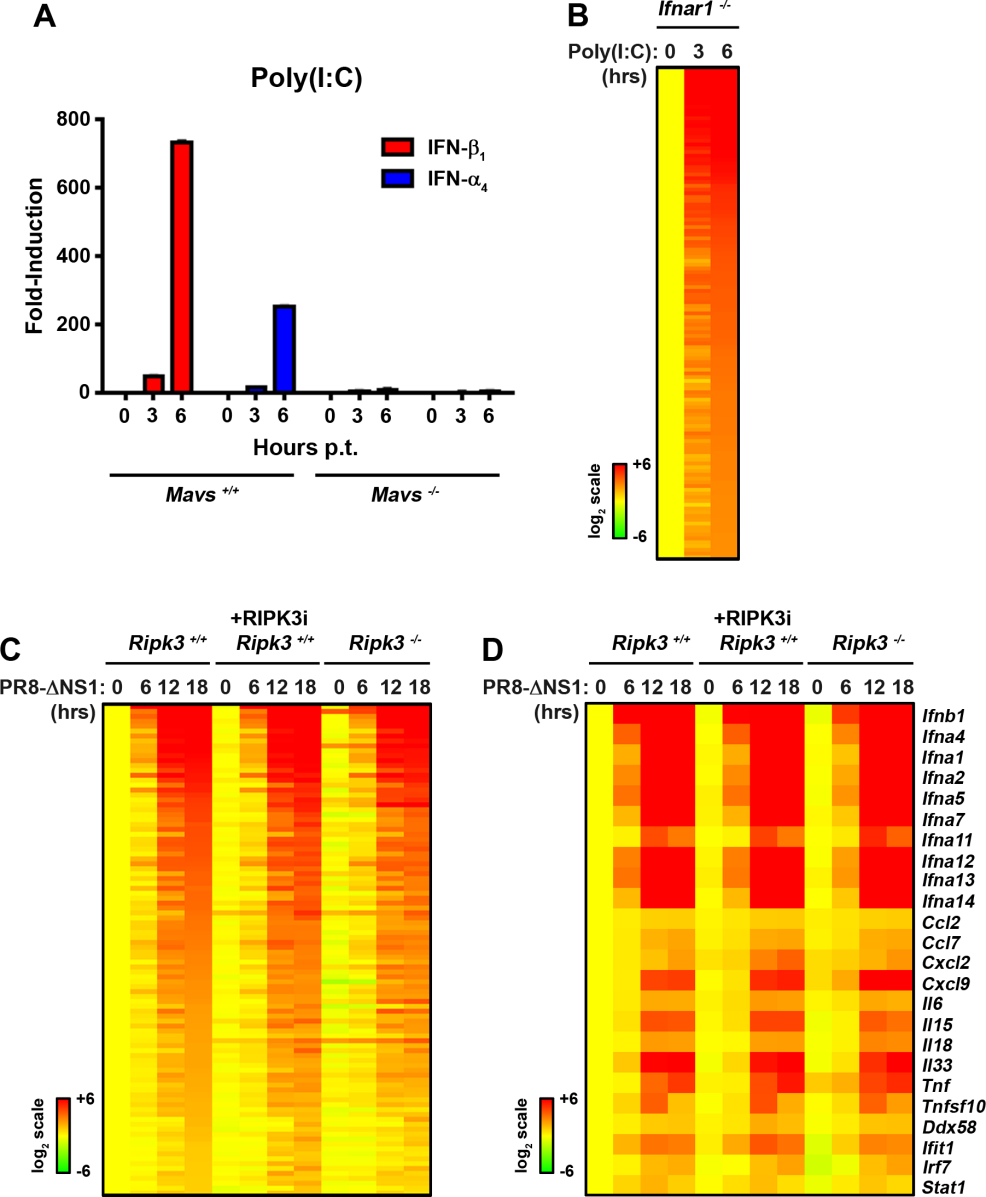
Role of RIPK3 in IAV-elicited RLR transcriptional responses. **(A)** qPCR determination of *ifnb1* and *ifna4* gene expression in *mavs*^*+/+*^ and *mavs*^*-/-*^ MEFs demonstrates that transfected poly(I:C) activates antiviral gene expression predominantly via the RLR pathway. **(B)** Heatmap showing expression profiles of genes upregulated by poly(I:C) in *ifnar*^*-/-*^ MEFs. Expression levels in untreated cells were normalized to one (2^0^, yellow), and genes demonstrating at least 6-fold induction at 6 h were designated the ‘core RLR transcriptomic signature’. Heat bars shown to the left represent relative expression levels on a log_2_ scale. Genes were sorted based on fold-induction at 6 h. **(C)** Heatmap displaying the behavior of core RLR transcriptomic signature in *ripk3*^*+/+*^ MEFs (columns 1–4), *ripk3*^*+/+*^ MEFs treated with RIPK3 inhibitor (GSK’872 at 5μM, columns 5–8), or *ripk3*^*-/-*^ MEFs (column 9–12) following infection with PR8-ΔNS1 (m.o.i. = 1). Expression levels in mock infected in *ripk3*^*+/+*^ MEFs were normalized to one (2^0^, yellow) and genes displaying at least two-fold upregulation at 18 h were considered IAV-inducible; none of these were found to be RIPK3 dependent. Genes are sorted based on fold-induction at 18 h in *ripk3*^*+/+*^ MEFs. **(D)** Heatmap showing expression profiles of genes encoding type I IFNs, inflammatory cytokines and chemokines after infection with PR8-ΔNS1 in *ripk3*^*+/+*^ MEFs, *ripk3*^*+/+*^ treated with RIPK3 inhibitor (GSK’872, 5μM), or *ripk3*^*-/-*^ MEFs. Expression levels in mock infected in *ripk3*^*+/+*^ MEFs were normalized to one (2^0^, yellow) Heat bar, depicting relative expression levels on a log_2_ scale, is shown to the left.

To identify direct RLR targets without interference from secondary IFN signaling, we employed early-passage murine embryo fibroblasts (MEFs) from type I IFN receptor-deficient (*ifnar1*^*-/-*^) mice. We triggered RLRs by transfecting *ifnar1*^*-/-*^ MEFs with the virus-mimetic poly(I:C), a well-established RLR stimulator when delivered into the cytosol [23]. We preferred poly(I:C) over live virus for these studies, as actively-replicating viruses may trigger several (e.g. stress-response) pathways in addition to RLRs. We first confirmed that transfected poly(I:C) signals primarily via RLRs in MEFs (rather by TLR3- or PKR-dependent mechanisms [24,25] for example) by demonstrating that induction of *ifnβ* and *ifna4* occurred in wild-type (*mavs*^*+/+*^), but not in RLR signaling-deficient *mavs*^*-/-*^ MEFs (Fig 1A). Next, we transfected *ifnar1*^*-/-*^ MEFs with the same dose of poly(I:C) and subjected these cells to whole-genome microarray analysis, as previously described [26]. We identified a total of 131 genes that were induced at least six-fold after 6 h of stimulation by poly(I:C), which we designated the core RLR transcriptomic signature (Fig 1B; S1 Table).

Having defined this signature, we next tested the contribution of RIPK3 to RLR-activated transcriptional responses in IAV-infected fibroblasts. We used in these experiments a mutant of PR8 (PR8-ΔNS1) that lacks the RLR signaling inhibitor NS1 and therefore does not dampen host antiviral gene transcription [27]. We infected confluent monolayers of primary, early-passage MEFs from *ripk3*^*-/-*^ and control *ripk3*^*+/+*^ mice with PR8-ΔNS1 over a time course of 18 h, and subjected RNA from these cells to microarray analyses. As RIPK3 may participate in gene transcription programs independently of its kinase activity, we also included in this analysis MEFs from *ripk3*^*+/+*^ mice that were treated with the RIPK3 inhibitor GSK’872 [19] before infection with PR8-ΔNS1. GSK’872 efficiently blocks RIPK3-driven necroptosis upon IAV infection [14] or TNF-α stimulation [19] in murine fibroblasts.

We found that, of the 131 genes induced six-fold or more by poly(I:C) in *ifnar1*^*-/-*^ MEFs, 100 genes (~80%) were induced two-fold or more in *ripk3*^*+/+*^ MEFs by PR8-ΔNS1, none of which were aberrant in their induction in *ripk3*^*-/-*^ MEFs (Fig 1C; S2 Table). Notably, induction of genes encoding type I IFNs (IFN-β and several IFN-α subtypes), chemokines and pro-inflammatory cytokines, including IL-6, IL-18, IL-33, TNF-α and TRAIL, or RLR signaling proteins (RIG-I, MDA-5, IRF7, and STAT1), did not require RIPK3 (Fig 1D; S3 Table). These results demonstrate that RIPK3 does significantly not contribute to IAV-activated RLR-driven transcriptional responses in MEFs. Of note, a small subset of 21 genes were upregulated (18) or downregulated (3) two-fold or more upon exposure of *ripk3*^*+/+*^ MEFs to GSK’872 alone, in the absence of any additional stimulus (S1 Fig). Some of these genes were also upregulated (11) or downregulated (3) 1.5-fold or more in untreated *ripk3*^*-/-*^ MEFs, suggesting they may be under basal RIPK3 control.

### Modest post-transcriptional defects in production of IFN-β in *ripk3*^*-/-*^ MEFs

**Fig 2:**
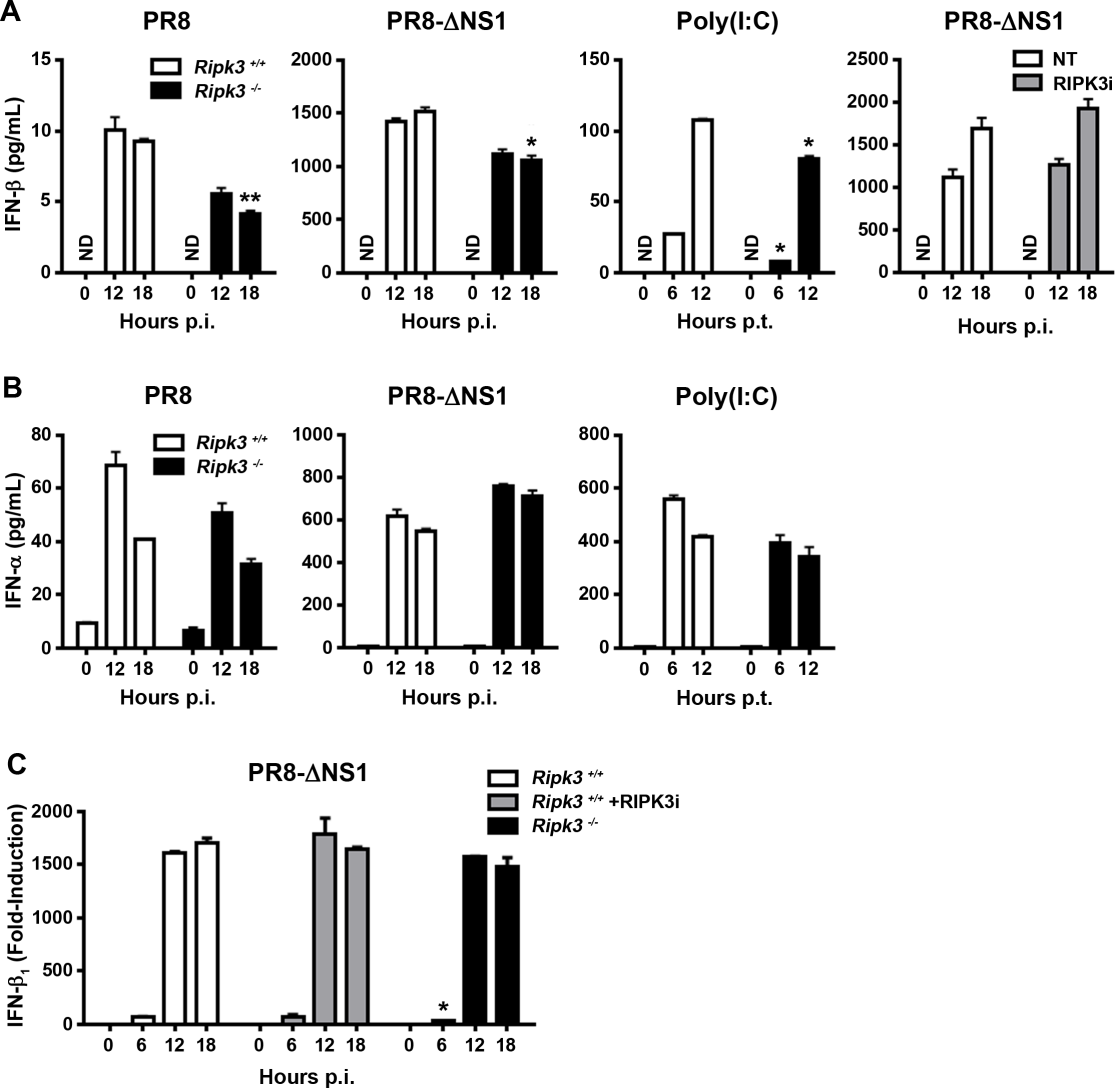
Modest post-transcriptional role for RIPK3 in production of IFN-β upon RLR stimulation. **(A)** *Ripk3*^*+/+*^ or *ripk3*^*-/-*^ MEFs were infected with WT PR8 (m.o.i. = 2), PR8-ΔNS1 (m.o.i. = 1), or transfected with virus mimetic poly(I:C) for the indicated times, and IFN-β in supernatants of cultured cells was quantified by ELISA. *Ripk3*^*+/+*^ MEFs were infected with PR8-ΔNS1 in the presence or absence of the RIPK3 kinase inhibitor GSK’872 (5μM), for the indicated times, and secretion of IFN-β in supernatants of cell culture was quantified by ELISA. **(B)** IFN-α in supernatants of cell culture was also quantified by ELISA. **(C)** *Ripk3*^*+/+*^ MEFs treated with or without RIPK3 inhibitor (GSK’872, 5μM), or *ripk3*^*-/-*^ MEFs, were infected with PR8-ΔNS1 (m.o.i. = 1), for the indicated times, and *ifnb1* mRNA levels determined from DNA microarray data output. Fold-induction was calculated using basal levels (time = 0) of each condition as baseline, which was normalized to 1. Error bars represent mean +/- S.D. NS = not statistically significant; ** *p* <0.005; * *p* <0.05.

We next examined type I IFN protein production from *ripk3*^*+/+*^ and *ripk3*^*-/-*^ MEFs after infection with WT PR8 or PR8-ΔNS1 or transfection with poly (I:C). Expectedly, PR8 induced very little IFN-β in *ripk3*^*+/+*^ MEFs, while PR8-ΔNS1 and poly(I:C) all triggered robust IFN-β production from these cells (Fig 2A). Interestingly, IFN-β production was modestly, but reproducibly, diminished in *ripk3*^*-/-*^ MEFs after infection with virus or transfection with poly (I:C) (Fig 2A). A RIPK3 inhibitor did not blunt IFN-β production from wild-type MEFs, arguing against a role for the kinase activity of RIPK3 in IFN-β synthesis (Fig 2A, right). As production of IFN-α was not significantly altered by loss of RIPK3 (Fig 2B), and as induction of *ifnb1* mRNA (encoding IFN-β) after infection with PR8-ΔNS1 was mostly indistinguishable between *ripk3*^*+/+*^ and *ripk3*^*-/-*^ MEFs (Fig 2C), these results suggest a modest post-transcriptional, kinase-independent role for RIPK3 in selective production of IFN-β after RNA virus infection of fibroblasts.

### RIPK3 is largely dispensable for the host antiviral transcriptional response to IAV in MEFs

**Fig 3:**
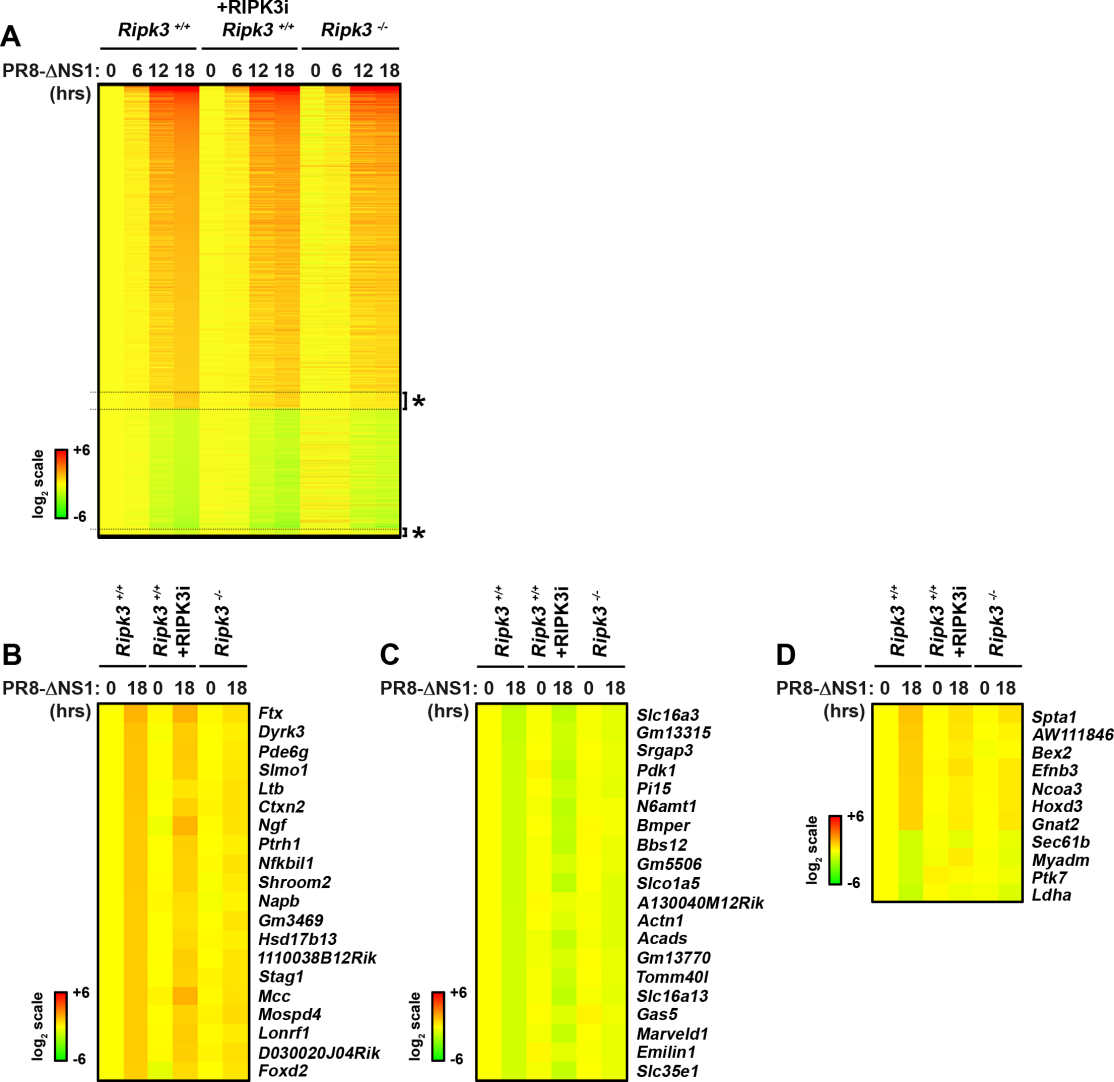
Role of RIPK3 in IAV-activated transcriptional responses. **(A)** Heatmap depicting behavior of IAV-regulated genes in *ripk3*^*+/+*^ MEFs (columns 1–4) or *ripk3*^*+/+*^ MEFs treated with the RIPK3 inhibitor (GSK’872 at 5μM, columns 5–8) or *ripk3*^*-/-*^ MEFs (columns 9–12) following PR8-ΔNS1 infection for the indicated times. All genes displaying two-fold or more change in expression following infection in *ripk3*^*+/+*^ MEFs at 18 h were considered IAV regulated. Expression levels in mock infected in *ripk3*^*+/+*^ MEFs were normalized to one (2^0^, yellow), and heat bars shown to the left represent relative expression levels on a log_2_ scale. RIPK3-dependent genes, defined as those IAV targets which were (i) less than 1.5-fold differentially expressed in *ripk3*^*-/-*^ MEFs at 18 h and (ii) less than 1.two-fold differentially expressed in untreated *ripk3*^*-/-*^ MEFs at 18 h, are clustered together and indicated by asterisks. **(B)** Heatmap showing the top 20 genes whose upregulation by PR8-ΔNS1 is dependent on RIPK3. **(C)** Heatmap showing the top 20 genes whose downregulation by PR8-ΔNS1 is dependent on RIPK3. **(D)** Heatmap showing all the genes whose up- or downregulation by PR8-ΔNS1 requires the kinase activity of RIPK3.

To test if RIPK3 regulated IAV-elicited RLR-independent gene-induction pathways, we examined the behavior of all genes induced by PR8-ΔNS1 in wild-type MEFs that were either untreated or were treated with the RIPK3 kinase inhibitor GSK’872 after infection, versus *ripk3*^*-/-*^ MEFs. Fort this analysis, we used a less-stringent cut-off (two-fold) to capture as many IAV-regulated genes as possible. A total of 1215 genes were found to be upregulated in wild-type MEFs two-fold or more by PR8-ΔNS1 18 h post-infection, and a further 475 genes were downregulated by the same factor at this time point, for a total of 1690 (Fig 3A, S4 Table). Of the genes upregulated by PR8-ΔNS1, only 59 were dependent on RIPK3 expression, of which seven were also dependent on its kinase activity. Of the genes whose transcription is downregulated following PR8-ΔNS1 infection, 28 were dependent on RIPK3, including four on its kinase activity (Fig 3D). The top 20 genes whose induction or downregulation by IAV are dependent on RIPK3 are shown in Fig 3B and 3C, and the 11 genes also requiring the kinase activity of RIPK3 are presented in Fig 3D; gene expression profiles for all these genes are shown in S4 Table. Attempts to further cluster the relatively small number (<100) of RIPK3-regulated genes into functional categories by standard *in silico* pathways enrichment approaches (e.g., Ingenuity Pathways Analysis) did not result in the identification of any relevant biological theme. As RIPK3-regulated genes, representing just ~5% of the IAV-induced transcriptome, are only modestly induced or downregulated by IAV in the first place, and as none of the identified genes were found to be obligate RIPK1 targets (i.e., completely dependent on RIPK3 for their regulation by IAV infection), these findings are collectively indicative of a minimal role, if any, for RIPK3 in the host transcriptional response to IAV in murine fibroblasts.

### RIPK3 is not essential for type I IFN signaling in MEFs

**Fig 4:**
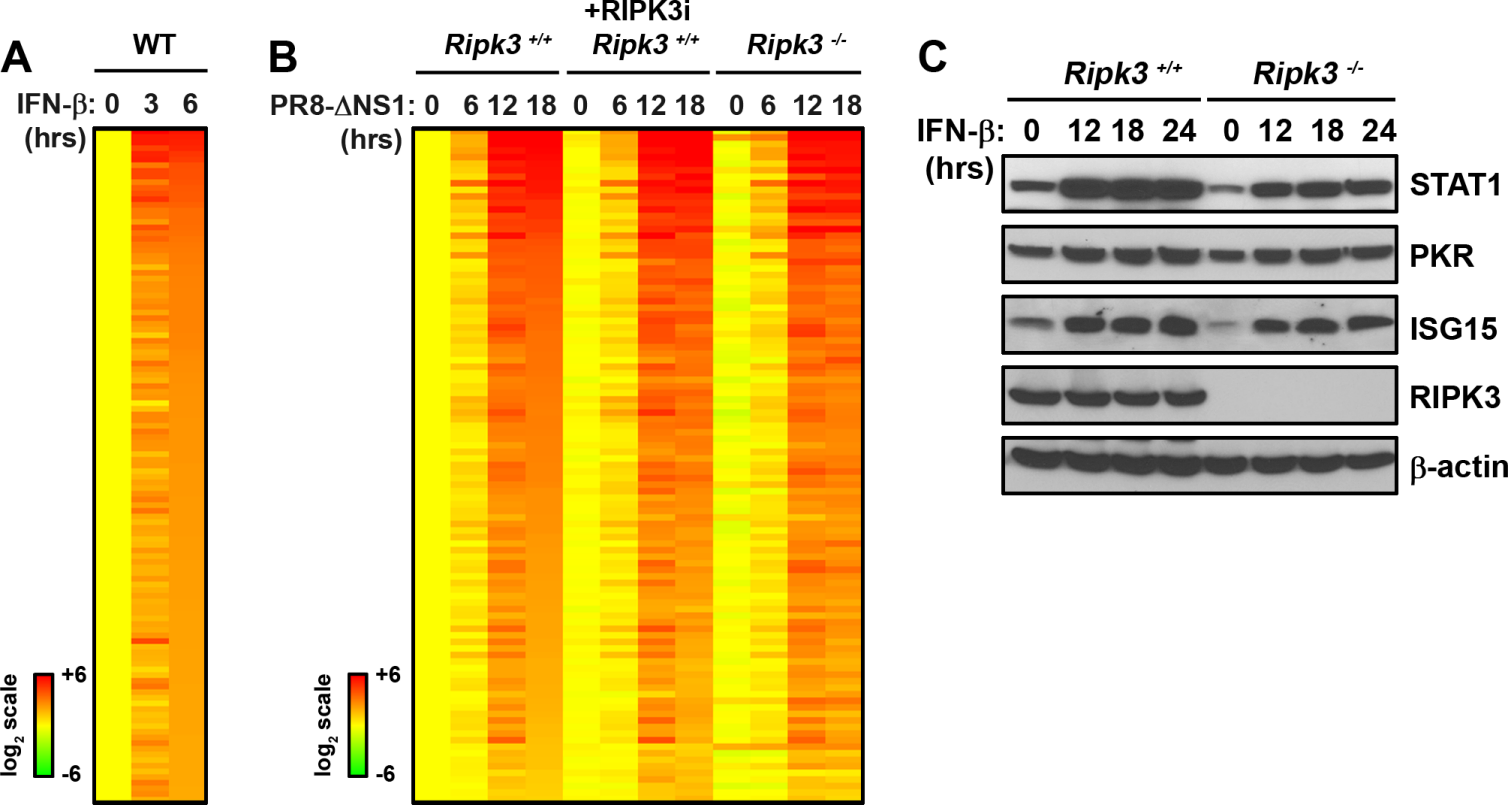
Normal IFN-β signaling in the absence of RIPK3. **(A)** Heatmap showing expression profiles of genes upregulated by IFN-β in WT MEFs. Expression levels in untreated cells were normalized to one (2^0^, yellow), and genes demonstrating 4-fold or more induction at 6 h were considered induced and sorted based on fold-induction at 6 h. Heat bars shown to the left represent relative expression levels on a log_2_ scale. **(B)** Heatmap displaying the behavior of IFN responsive genes in *ripk3*^*+/+*^ MEFs (column 1–4), *ripk3*^*+/+*^ MEFs treated with RIPK3 inhibitor (GSK’872 at 5μM, column 5–8), or *ripk3*^*-/-*^ MEFs (column 9–12) following PR8-ΔNS1 infection. Expression levels in mock infected in *ripk3*^*+/+*^ MEFs were normalized to one (2^0^, yellow) and genes displaying at least two-fold changes at 18 h were considered IAV regulated. Genes are sorted based on fold-induction at 18 h in *ripk3*^*+/+*^ MEFs. No genes were found to be dependent on RIPK3. **(C)** Levels of ISG-encoded proteins were compared in *ripk3*^*+/+*^ and *ripk3*^*-/-*^ MEFs by immunoblot analysis following treatment with IFN-β (1000U/mL) for the indicated times.

To test if RIPK3 was involved in type I IFN signaling, we first identified the core type I IFN-activated transcriptome in MEFs. 119 genes were found to be upregulated at least four-fold by IFN-β at 6 h (Fig 4A). None of these genes were found to be dependent on RIPK3, or its kinase activity, for their induction by IAV. To confirm these findings at the protein level, we show that three representative ISG products, STAT1, PKR, and ISG15, are all induced equivalently in *ripk3*^*+/+*^ and *rip3*^*-/-*^ MEFs (Fig 4C). Modest differences in basal protein levels of some ISGs (e.g., STAT1, ISG15) were noted, but these differences were not reproducible. Altogether, these findings reveal that RIPK3 does not appreciably contribute to type I IFN-activated transcriptional responses during IAV virus infection in MEFs.

## Discussion

This study was designed to interrogate the role of RIPK3 in IAV-activated transcriptional responses in general, and the RLR response in particular. We find little evidence for a role for RIPK3 in the host antiviral transcriptional response to IAV in MEFs. Only ~5% of the IAV-induced transcriptome was affected to any significant extent by the loss of RIPK3, and none of the affected genes were obligate RIPK3 targets (i.e., completely dependent on RIPK3 for change in their expression levels upon infection). Primary RLR target genes, including type I IFNs and other critical pro-inflammatory chemokines and cytokines, were mostly unaffected at the transcriptional level by loss of RIPK3.

Surprisingly, IFN-β expression was modestly attenuated by RIPK3 loss, perhaps at a post-transcriptional step. It is possible that RIPK3 may control secretion of IFN-β protein, or that the lysis of the infected cell is necessary for efficient release of IFN-β. Alternatively, RIPK3 may regulate *ifnb1* transcription either basally or very early after infection, like NF-κB does [28], resulting in a temporal delay in *ifnb1* induction that was not immediately revealed by our DNA microarray analysis but may manifest as a modest defect in IFN-β production later in the course of the infection. Whether RIPK3 controls basal/early transcription of the *ifnb1* gene, contributes to the stability of *ifnb1* mRNA, regulates its translation, and/or mediates IFN-β secretion remains to be determined.

Our previous work has revealed a role for RIPK3-driven cell death in controlling an IAV infection *in vivo* [14]. We have found that RIPK3 activates parallel pathways of MLKL-driven necroptosis and FADD-mediated apoptosis to limit IAV replication. In the absence of RIPK3, virus spread and progeny virion production are both increased in the infected *ripk3*^*-/-*^ lung, compared to controls [14]. As we and others have previously found that even small differences in the timing and magnitude of IFN-β production can affect the control of an acute RNA virus infection, it is conceivable that the modest defects in IFN-β production seen upon IAV infection of RIPK3-deficient fibroblasts may contribute to the susceptibility of *ripk3*^*-/-*^ mice, especially when cell-death pathways of virus clearance are compromised and non-cytolytic means of virus control, such as those activated by type I IFNs in uninfected neighboring cells, become critical to halting virus spread.

It will be interesting to determine the contribution of RIPK3 to the type I IFN response in specialized IFN producing cell-types, as patrolling alveolar macrophages (which are the dominant producers of IFN-α in the virus-infected lung [29]) or plasmacytoid DCs (which rely on non-RLR-based sensing mechanisms for induction of type I IFNs).

## Supporting Information

S1 Fig Heatmap showing expression profile of genes upregulated or downregulated two-fold or more in uninfected *ripk3*^*+/+*^ MEFs upon exposure to the RIPK3 kinase inhibitor GSK’872. Also shown is the behavior of these genes in uninfected, untreated *ripk3*^*-/-*^ MEFs. Related to Figs 1 and 2.
(TIF)

S2 Fig Uncropped scans of immunoblots. Related to Fig 4C.
(TIF)

S1 Table List of all 131 genes upregulated in *ifnar1*^*-/-*^ MEFs after poly(I:C) treatment for 6 h. Related to Fig 1B.
(XLSX)

S2 Table Behavior of genes upregulated in *ifnar1*^*-/-*^ MEFs by poly(I:C) treatment (S1 Table) in *ripk3*^*+/+*^ MEFs or *ripk3*^*+/+*^ MEFs treated with RIPK3 inhibitor or *ripk3*^*-/-*^ MEFs following RP8-ΔNS1 infection for 18 h are shown. Related to Fig 1C.
(XLSX)

S3 Table List of selected type I interferon genes and related genes after PR8-ΔNS1 infection in *ripk3*^*+/+*^ MEFs or *ripk3*^*+/+*^ MEFs treated with RIPK3 inhibitor or *ripk3*^*-/-*^ MEFs. Related to Fig 1D.
(XLSX)

S4 Table List of all 1690 genes regulated by infection with PR8-ΔNS1 in *ripk3*^*+/+*^ MEFs or *ripk3*^*+/+*^ MEFs treated with RIPK3 inhibitor or *ripk3*^*-/-*^ MEFs are shown. Top 20 upregulated or downregulated genes dependent on RIPK3 are colored in red or green, respectively. In addition, genes upregulated or downregulated dependently on RIPK3 kinase activity are colored in pink or light green, respectively. Related to Fig 3A and 3B and 3C and 3D.
(XLSX)

S5 Table List of all 119 genes upregulated in WT MEFs after IFN-β treatment at 6 h. Related to Fig 4A.
(XLSX)

S6 Table Behavior of genes upregulated in WT MEFs by IFN-β treatment (S5 Table) in *ripk3*^*+/+*^ MEFs or *ripk3*^*+/+*^ MEFs treated with RIPK3 inhibitor or *ripk3*^*-/-*^ MEFs following PR8-ΔNS1 infection for 18 h. Related to Fig 4B.
(XLSX)
