# Supplementary figures and images for: RIPK3 Is Largely Dispensable for RIG-I-Like Receptor- and Type I Interferon-Driven Transcriptional Responses to Influenza A Virus in Murine Fibroblasts
Source: PLoS One. 2016 Jul 8;11(7):e0158774. doi: 10.1371/journal.pone.0158774 (PMC4938532; doi:10.1371/journal.pone.0158774)

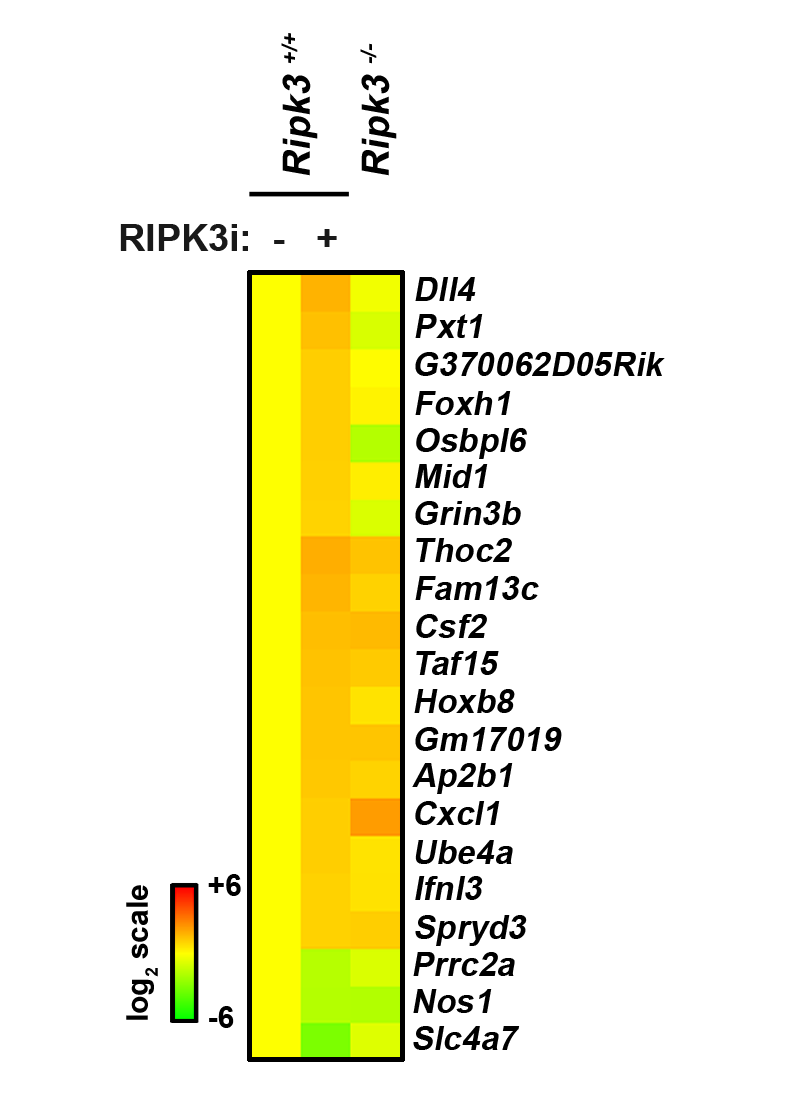

Supplement: S1 Fig — Also shown is the behavior of these genes in uninfected, untreated ripk3-/- MEFs. Related to Figs 1 and 2. (TIF) [file pone.0158774.s001.tif]

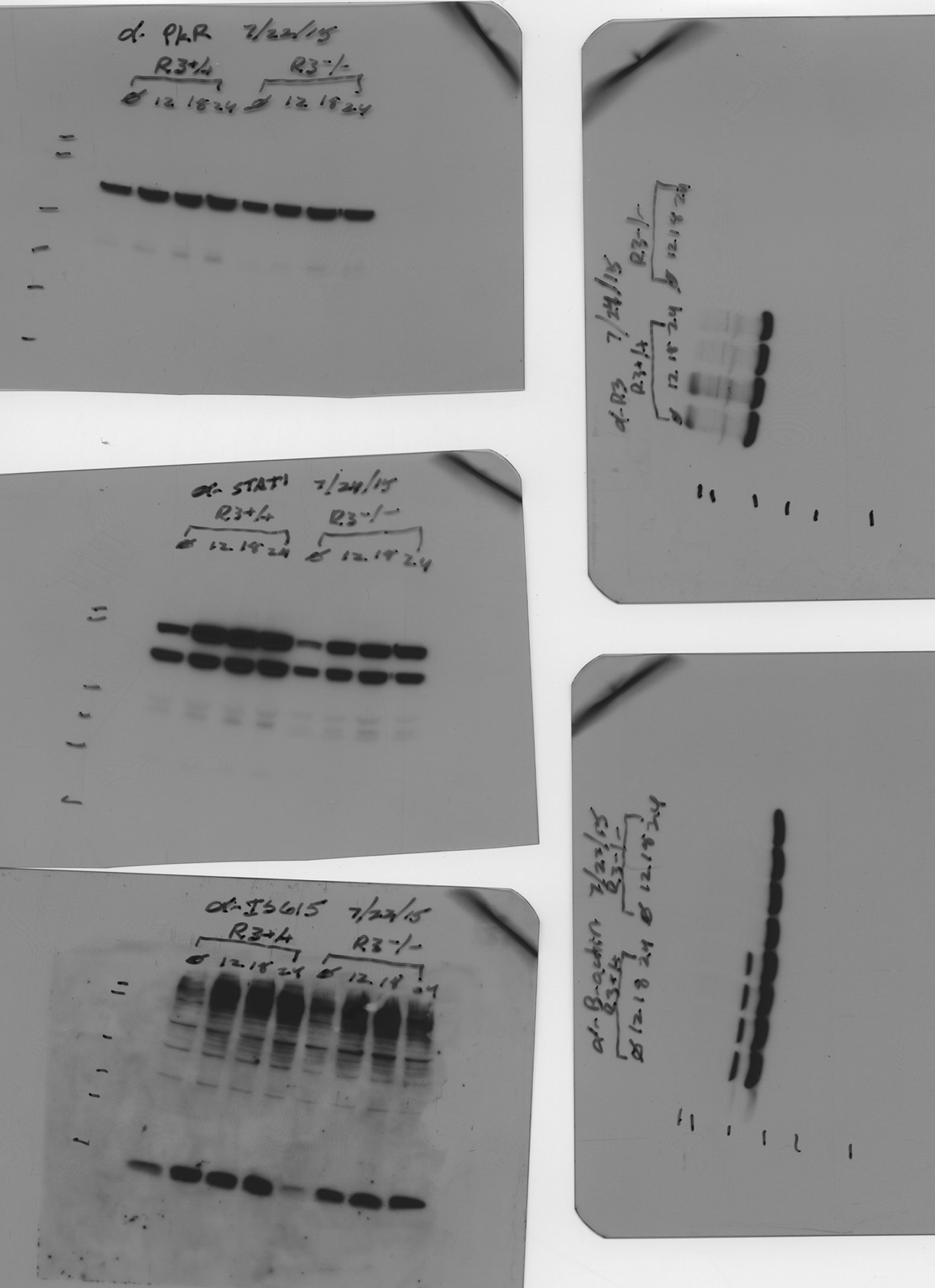

Supplement: S2 Fig — Related to Fig 4C. (TIF) [file pone.0158774.s002.tif]
